# Methodology of an International Study of People with Multiple Sclerosis Recruited through Web 2.0 Platforms: Demographics, Lifestyle, and Disease Characteristics

**DOI:** 10.1155/2013/580596

**Published:** 2013-04-11

**Authors:** Emily J. Hadgkiss, George A. Jelinek, Tracey J. Weiland, Naresh G. Pereira, Claudia H. Marck, Dania M. van der Meer

**Affiliations:** ^1^Emergency Practice Innovation Centre, St Vincent's Hospital, Melbourne, VIC 3065, Australia; ^2^Department of Epidemiology and Preventive Medicine, Monash University, Melbourne, VIC 3004, Australia; ^3^Department of Medicine, The University of Melbourne (St Vincent's Hospital), Melbourne, VIC 3065, Australia; ^4^Faculty of Medicine, The University of Notre Dame Australia, Fremantle, WA 6959, Australia

## Abstract

*Background*. Despite evidence of the potential importance of the role of health and lifestyle behaviours in multiple sclerosis (MS) outcomes, there has not been a significant focus on this area of research. *Aim*. We aimed to recruit an international sample of people with MS at baseline and over a five-year timeframe, examine their health and lifestyle behaviours, and determine the relationship of these behaviours to self-reported disability, disease activity, and quality of life. *Methods*. People with MS were recruited through web 2.0 platforms including interactive websites, social media, blogs, and forums and completed a comprehensive, multifaceted online questionnaire incorporating validated and researcher-derived tools. *Results*. 2519 participants met inclusion criteria for this study. This paper describes the study methodology in detail and provides an overview of baseline participant demographics, clinical characteristics, summary outcome variables, and health and lifestyle behaviours. The sample described is unique due to the nature of recruitment through online media and due to the engagement of the group, which appears to be well informed and proactive in lifestyle modification. *Conclusion*. This sample provides a sound platform to undertake novel exploratory analyses of the association between a variety of lifestyle factors and MS outcomes.

## 1. Introduction

Multiple sclerosis (MS) is a chronic, debilitating neurological condition that affects an estimated two million people worldwide. Despite decades of research, the aetiology of MS and factors that affect disease progression and relapse rate are still debated [1]. Symptoms of the disease are heterogeneous, and the current goal of therapeutic intervention is to slow the progression of disability and provide symptomatic relief.

Several large national MS registries collect longitudinal data on patient outcomes and measure the effectiveness of a range of therapies [2]. Epidemiological studies that utilise these data are a cost-effective way of exploring associations between factors that influence relapse rate or disease progression. There is a comprehensive body of the literature supporting a relationship between lifestyle and psychosocial factors, and MS health outcomes [3–8]. Despite this expanding body of research, little is known about the degree to which beneficial lifestyle modification is adopted by the international MS community and whether this has a positive impact on health outcomes. To our knowledge, none of these registries collect comprehensive data on lifestyle factors. 

We aimed to recruit followers of online media engaging people with MS and examine in detail their health and lifestyle behaviours and determine the relationship of these factors to self-reported disability, disease activity, and quality of life, with a follow-up period of five years. For many people with chronic diseases, the internet is an important tool for self-education, emotional support, and practical advice from people facing similar challenges [9]. Web 2.0 platforms like Facebook, Twitter, forums, and blogs are powerful tools that enable patients to develop greater self-efficacy [10]. Specifically, MS resources encompass an extensive online presence [11]. We aimed to recruit people to our study through these platforms as this enabled us to gather a sample of people with MS containing many who are making lifestyle and other risk-modifying changes, thereby allowing comparison between patients with a range of different approaches to self-management. We also suspected that many online patients are highly proactive and have an interest in self-help and lifestyle modification, offering a different perspective to the more traditional approaches to studying treatment, currently assessed in national registries. 

This study provides a snapshot of current lifestyle and risk-modifying behaviours of a large international group of people with MS, as well as an ongoing platform for analysing the association between these variables and disease progression, that have not previously been examined in detail. They will also help to inform future research, and people with MS, of the potential contribution of lifestyle to their health-related quality of life, disease activity, and physical disability. This paper reports in detail the methodology of the study and an overview of the characteristics of participants recruited. Future studies will seek to analyse and report associations between lifestyle variables and disease data in detail.

## 2. Materials and Methods

### 2.1. Participants and Data Collection

This survey, collecting the baseline data for the health outcomes and lifestyle interventions in a sample of people with multiple sclerosis (HOLISM) study, was conducted using the online software SurveyMonkey. A webpage was created, inviting people to take part in the study, with a description of the study aims. Online recruitment for the study took place over a 15-week period, using websites, a mailing list, and web 2.0 platforms such as blogs, forums, Facebook, and Twitter. The principle investigator (GJ) had developed an online presence in the MS community over the 12 years prior to the study due to his extensive work advocating for lifestyle modification and integrated MS management, including moderating a website dedicated to this field (http://www.overcomingmultiplesclerosis.org; “OMS”). Two members of the research team (GJ and EH) systematically identified Facebook groups and pages with over 500 members or followers, designed for people with MS. On a weekly, or biweekly basis (depending on site traffic), the researchers invited people with MS to take part in the study and posted a link to the survey. Members of the OMS website were informed of the study by email and invited to participate. This was followed up with several reminders over the course of the recruitment period. Moderators of several popular MS blogs were contacted and asked to post information about the survey on their websites. Several MS societies were emailed and asked to promote the study through their websites or social media. The response to these requests was positive, and many people continued to voluntarily promote the study through their own online networks, contacts, and communities, creating a snowball effect.

The survey webpage linked individuals to a participant information sheet, which they were asked to read before giving consent. This was required for continued participation in the survey. Anyone formally diagnosed with MS by a medical doctor was encouraged to take part, but participants were excluded if they were under 18 years of age. This was verified in the survey: if the year of birth selected indicated that they were under 18 years of age, skip logic took the participant to the end of the survey. All participants were required to complete their contact details to facilitate followup. Data were stored in a reidentifiable form, and data security measures were undertaken to ensure that only members of the research team had access to participant information. The research team was available to answer participants' questions by phone and email. Ethics approval was granted by St Vincent's Hospital Melbourne Human Research Ethics Committee (LRR 055/12). 

### 2.2. Data Collection and Tools

The survey consisted of a maximum of 163 questions, taking around 40 minutes to complete, although skip logic enabled participants to avoid some questions not relevant to them. Three members of the research team searched and reviewed validated tools for use in the survey. Where possible, a tool was chosen that was psychometrically sound and had been tested in a similar study (Table 1). Some questions were unique to the domains assessed, in particular lifestyle variables that had not been extensively investigated, and hence validated questions or tools were not available. These included items on sun exposure, vitamin D supplementation, Omega-3 supplementation, and meditation and stress reduction practices. Responses were usually multiple choice and categorised to reduce respondent error through free-text numerical response. Such items were developed by the research team with consideration of studies that examined similar outcome variables. For any anthropometric or other measures, units utilised across different countries were considered. Although participants were encouraged to answer all questions, only completion of contact details was compulsory. Many of the items had been piloted already with a cohort of people with MS (manuscript in preparation) related to a longitudinal study [6]. The survey was piloted online with seven people with MS, and minor alterations were made based on their feedback.

The survey consisted of the following domains.

*Sociodemographics*. This domain consisted of items collecting data on age, gender, current location, country of birth, parents' countries of birth, cultural background, marital status, number of children, employment, education, height, and weight. All sociodemographic items were multiple choice. Cultural background was categorised according to the Australian standard classification of cultural and ethnic groups [20], with an additional free-text “other” response available. 

*Diagnostic History*. This included confirmation of MS diagnosis by a medical doctor, year of diagnosis, first year of symptoms, diagnostic investigations undertaken, number of brain and spinal lesions, and subtype of MS on diagnosis/currently. Some of these items were modified from the North American Research Committee on Multiple Sclerosis (NARCOMS) enrolment questionnaire. Although only people with a formal diagnosis of MS were invited to participate, to improve the validity of self-report, those who could not confirm their diagnosis but had clinically isolated syndrome or “possible MS” were still able to complete the questionnaire. However, for the purpose of this paper, analyses were only performed for those self-reporting a confirmed diagnosis of MS.

*Disease Activity*. This was determined through participant's reported relapse rate (self-diagnosed using the definition of a relapse as provided in the NARCOMS enrolment questionnaire; and physician-diagnosed) in the last 12 months and the last 5 years; number of relapses treated with steroids and whether currently experiencing symptoms due to recent relapse. A five-year annualised relapse rate was derived by dividing the number of relapses over five years by the number of years of disease with an upper limit of five. 

*Level of Disability.* The patient-determined disease steps (PDDS; Table 1) is a self-reported surrogate tool to the expanded disability status scale (EDSS) which is commonly used by neurologists to assess gait disability [12]. It is scored ordinally from 0 (normal) to 8 (bed bound). It is simple and easy to administer, correlates well with the EDSS and moderately with the widely used multiple sclerosis functional composite, and has excellent concordance between raters. It is also deemed a practical tool to use to assess changes in disability over time [21]. The PDDS has been used in a number of studies associated with the NARCOMS registry [22–24].

*Comorbidities*. The self-administered comorbidity questionnaire (SCQ) is an efficient method of assessing the presence of comorbidities in the absence of medical record review [13]. It also determines whether treatment is received and if the condition limits activities. It correlates modestly with the Charlson comorbidity index and has been used in a study of participants with MS [25]. For the purpose of this study, two arthritic conditions were combined into one, and due to anticipated high prevalence, anxiety was also listed as a condition.

*Health-Related Quality of Life*. The multiple sclerosis quality of life-54 (MSQOL-54) is a measure of health-related quality of life (HRQOL) that was developed from the RAND 36-item health survey (SF-36) and supplemented with 18 additional items. The MSQOL-54 consists of 52 items distributed into 12 scales and two single items, which give rise to two composite scores—the physical and mental health composites. Internal consistency reliability estimates for the 12 multi-item scales ranged from 0.75 to 0.96 in a sample of 179 patients with multiple sclerosis [14]. Test-retest intraclass correlation coefficients ranged from 0.66 to 0.96, and construct validity has been shown to be strong. The tool has since been extensively validated and translated in international populations [26–28], and in assessing the impact of fatigue [29], depression [30], and sexual dysfunction [31], as well as a number of medical therapies.

*Dietary Habits*. The diet habits questionnaire (DHQ) is a 22-item dietary assessment tool, validated in an Australian cardiac disease population [15]. It assesses saturated and nonsaturated fat intake, fruit and vegetable, fibre, takeaway, snack habits, and omega-3 consumption, among other estimates. To reduce respondent burden, we elected for a brief dietary screening tool over a more elaborate food diary or food frequency questionnaire. Shorter, nutrient-specific, semiquantitative questionnaires were not appropriate for our study, which aimed to assess diet more broadly. 

*Smoking and Alcohol*. Frequency and number of alcoholic drinks were measured with examples of a standard drink provided. Other items were current smoking status, number of cigarettes smoked, and year quit, if previously smoked. 

*Medication Use*. A list of 24 disease modifying drugs (DMDs) and other common MS medications including generic and trade names were provided, and participants were asked to indicate current and previous use, including length of time taken. In addition, participants were asked to indicate whether they took prescription, over-the-counter, or herbal agents for 10 symptomatic conditions: depression, anxiety, headaches, other pain, fatigue, difficulty sleeping, bladder problems, bowel problems, spasticity, and “other”. 

*Sunlight and Vitamin D*. A total of seven researcher-devised items explored participants' average weekly frequency of sun exposure, current vitamin D supplementation and dosage, serum vitamin D level, and recency of test. Adequate sun exposure was considered to be 10–15 minutes of sunlight on a day with UV index of 7 (more or less if the UV index is lower or higher).

*Omega-3*. Items included both the type and daily dosage of omega-3 supplementation used on average in the last 12 months. Types of omega-3 included fish oil, high strength fish oil, flaxseed oil, and “other” free-text responses.

*Exercise*. The international physical activity questionnaire (IPAQ) is a 7-day recall of frequency and duration of vigorous and moderate activity participation: walking and sitting [16]. Items can be scored separately, as a combined total score, or computed as metabolic equivalent of task (MET) minutes. It has been validated in a number of studies and population groups globally, including MS populations [32, 33]. 

*Stress Management*. Four researcher-devised items measured the weekly frequency and length of meditation practice undertaken on average over the previous 12 months, other stress reduction activities practiced by respondents (free-text option), and the self-reported efficacy of such activities, categorised.

*Social Support*. The single item measure of social support (SIMSS) was employed to determine the number of people that provided support to participants. This item was developed by Blake and McKay (1986) and is a predictor of morbidity among women [17]. Responses of 0 or 1 indicate a low tangible assistance; 2 or more indicate high tangible assistance. It has been used in multiple studies with cancer patients [34, 35].

*Fatigue*. The fatigue severity scale (FSS) consists of nine fatigue-related statements rated on a seven-point scale from “disagree” to “agree” [18]. It has good internal consistency, stability, and sensitivity to change over time and is frequently used to assess fatigue in MS populations [29, 36]. A mean score ≥ 4 has been suggested as a cutoff to indicate clinically significant fatigue and has been used in several other studies [36–38].

*Depression*. The patient health questionnaire (PHQ-9) is a depression screening tool which has been validated in patients with MS [39]. The patient health questionnaire depression module short version (PHQ-2), utilised in our study, is a two-item version of the PHQ-9 which has shown good construct and criterion validity in a sample of 6000 patients, with a reported sensitivity of 83% and specificity of 92% for major depression with a score ≥ 3 [19]. The PHQ-2 can be complemented by the emotional subscore of the MSQOL-54.

*Engagement*. Four items are asked about engagement in OMS resources and other sources of information on lifestyle and MS, such as books, websites, or educational programs (free-text response). 

### 2.3. Data Analysis

The statistical package for the social sciences (SPSS) version 20.0 was used to calculate statistics. Univariate analyses were performed and continuous data reported using mean (95% CI) or median (IQR) and categorical data using number and percentage. Due to variation in item completion, analyses were calculated using item response as the denominator. Where possible, summary scores from validated tools were derived according to scoring instructions or as suggested in the literature. The following are explanations of how new variables and summary scores were derived for the purpose of this paper.

A planned variable, “disease activity,” was derived from data reporting relapse rates (doctor diagnosed) for those with relapsing-remitting MS only. It was categorised as increasing, decreasing, or stable, where relapse rate in the preceding 12 months was higher, lower, or the same, respectively, as the 5-year annualised relapse rate. For the purpose of this paper, conditions listed in the SCQ were summed to determine the proportion of participants that had one or more comorbidity. “Other” free-text responses will be categorised and reported in a future study along with reporting of the treatment and limitations relevant to each condition. 

The MSQOL-54 was scored according to the scoring instructions with a set number of items required to be completed in order to give rise to the subscores, which in turn were required for calculation of the composite scores; hence, there was variability in the completion rates. 

Cutoff scores were set as an aggregate score ≥ 3 in the PHQ-2 to screen positive for major depression, and a mean score ≥ 4 in the FSS to indicate clinically significant fatigue, as defined in the literature. To derive both of these summary scores, full item completion was required. 

Based on recency of licensing and mode of action, the medications were grouped into seven categories (Table 2). Free-text responses for “other” medications were reviewed and recategorised into existing variables.

## 3. Results

**Figure 1:**
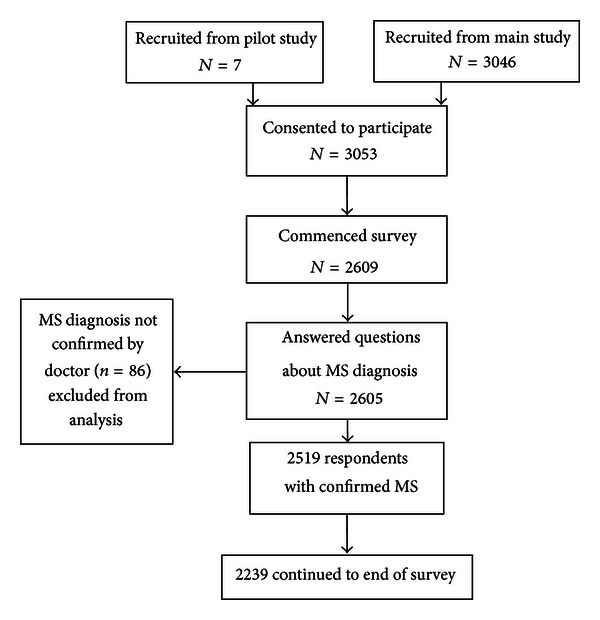
Participant study inclusion criteria.

A total of 3053 participants consented to participate, seven of whom were pilot study participants, whose data were included in analysis. Of those that consented, 2519 had been formally diagnosed with MS and therefore met the criteria for study inclusion. 2518 of these provided contact details for followup (Figure 1). Of those with confirmed MS, 89% continued to the end of the survey. Of the demographics and clinical characteristics items, there was an average item completion rate of 95%.

### 3.1. Sociodemographic Data

The majority of respondents were women, comprising 82.2% of the sample (Table 3). Participants' ages ranged from 18 to 87, with a median age of 46 years (IQR 38–53). Country of residence spanned the globe, with participants originating from 77 countries and living in 57 different countries at the time of the survey (Table 10). Respondents residing in the United States, Australia, United Kingdom, New Zealand, and Canada comprised 88% of the study sample. The majority of respondents (61.1%) were married, and over two-thirds had children. Around one-third were engaged in full-time work, whilst 23.4% had retired due to medical reasons or disability. Over half had completed a university bachelor's degree, and of these, 64.0% had also completed a postgraduate degree.

### 3.2. MS Diagnosis

A median of 13 years (IQR 7–21) had passed since participants first experienced symptoms of MS, whilst participants had formally been diagnosed with MS for a median of six years (IQR 3–12), with 45.0% having being diagnosed within the previous five years (Table 4). The median age at diagnosis was 37 (IQR 30–45). The majority of the sample had relapsing-remitting MS (61.0%), which was also the most common subtype at time of diagnosis (76.7%; Table 5).

### 3.3. Disease Progression

The level of disability, as measured with the PDDS, was across all disability spectrums, but 31.7% reported having no symptoms or mild symptoms that return to normal after an attack: “normal” (Table 4). The median PDDS score was 3.0 (IQR 1.0–5.0). At the time of the survey, over one-quarter of participants were experiencing symptoms due to a recent relapse. 

### 3.4. Relapse Rate

Over the previous 12 months, relapsing-remitting participants self-reported an average of 1.09 relapses, and over the last five years, they self-reported an average of 0.97 relapses per year (Table 6). This was higher than relapse rate diagnosed by a doctor. For those with relapsing-remitting MS (with sufficient data; *n* = 1382), based on diagnosis by a doctor, disease activity was decreasing for 42.0%, increasing for 31.0%, and remaining stable for 27.0%.

### 3.5. Quality of Life, Depression, and Fatigue

Median summary scores from the MSQOL-54 were 68.4 (IQR 55.0–81.65) for the overall quality of life subscore, 59.2 (IQR 42.3–77.2) for the physical health composite, and 72.0 (IQR 51.2–84.1) for the mental health composite (Table 7). Using a score of 3 as a cutoff in the PHQ-2, nearly one-fifth of the sample screened positive for depression (Table 8). Respondents had a median overall score on the FSS of 4.9 (IQR 3.2–6.1), and 65.7% (95% CI 63.7–67.7) screened positive for clinically significant fatigue. 

### 3.6. Health and Lifestyle

The following variables are a snapshot of participant responses and not comprehensive of all variables examined; these will be examined in greater detail in subsequent papers. 68.4% of participants reported having one or more of the listed comorbidities in the SCQ. At the time of the survey, 51.4% of respondents were taking a first or second generation DMD. According to the single item measure of social support, the majority (59.5%) had 2–5 people in their life that they could count on in times of difficulty, but 5.3% of the sample had no one. 38.0% of respondents did not consume dairy products, 26.7% did not consume meat products, and 21.5% consumed neither meat nor dairy. 11.7% were current smokers. Over two-thirds (67.0%) intentionally exposed themselves to the sun to try to raise their vitamin D levels, and 82.3% took vitamin D supplements. Nearly two-thirds of respondents (64.3%) took omega-3 supplements. Meditation practice was undertaken at least once per week by 676 respondents (30.0%). 

## 4. Discussion

This is the first cross-sectional study examining health and lifestyle behaviours in a large international sample of people with MS using web 2.0 platforms. Our results suggest that the online MS community is a unique sample to study. The proportion of women that participated is an overrepresentation from the estimated incidence of 1.8 female cases for every male [40]. Studies show that women are more likely to participate in online research [41], which may explain this finding. The age range in our sample suggests that engagement with online resources or research is not exclusively for young, technology-savvy individuals but rather is embraced by all age groups. The majority of participants in our study are highly educated, with a significant number having completed one or more tertiary degrees. This has important implications because it is well established that there is a strong gradient for education and health behaviour and health status [42]. More educated patients are more likely to have a higher level of health literacy and have the resources to seek out, understand, and apply information about prevention and treatment for their condition [42], including use of the internet. Among people with MS, a higher level of education is associated with greater involvement in health care [43] and greater health-related quality of life [44]. 

In general, participants of this study had been diagnosed recently and had a low level of disability. This concurs with results from a self-enrolling online registry—NARCOMS—that reported one-third of their participants enrolled within two years of diagnosis and also had a median disability score of 3 (1–5) on the PDDS [22]. Compared to the MSBase international registry (which is physician enrolled) with over 11,000 participants from 26 countries, in general the participants in our study were older at enrolment, were diagnosed at an older age, and had a shorter disease duration at enrolment [45] (Table 9). It is possible that patients who are recently diagnosed seek out information and support through online communities early in the illness and may be more motivated to contribute to research in the hope that it will offer some new evidence for the successful treatment of their condition. 

It has been noted that clinical outcome measures of relapse rate and disability are insufficient alone to measure the impact of MS as they do not reflect patients' experiences of the disease [46]. Measures of health-related quality of life, fatigue, and depression are integral to gain a broader picture of the complex, multifaceted nature of the disease. Around one-fifth of our sample screened positive for depression. Depression commonly affects people with MS, and the prevalence rate in our sample is similar to that of Patten et al. (2003), who reported a 12-month prevalence of major depression in 26% of their MS sample aged 18–45 [47]. A review of studies on depression in people with MS found that lifetime prevalence rates of about 40–50% and 12-month prevalence rates of around 20% are commonly reported [48]. Fatigue is known to be one of the most debilitating, but poorly understood symptoms of MS [36]. Our data indicate that a significant proportion of MS participants have clinically significant fatigue, but the median overall score was lower than that reported with another large cohort [38]. Further analysis of the data will be undertaken to investigate the relationship between quality of life, depression, fatigue, and other variables in the study. 

Wide-ranging health and lifestyle behaviours have not been studied extensively among people with MS [49]. A surprising number of participants in our study reported undertaking health and lifestyle behaviours not commonly observed among the general community and not previously reported with an MS sample, to our knowledge. This included diets containing no meat or dairy products, a high intake of omega-3 and vitamin D supplementation, lower smoking rates, and meditation practice, among other lifestyle modifications. Other studies have demonstrated high use of nonpharmacological therapies among people with MS [50–52], but the adoption of dietary practices, meditation, and vitamin D and omega-3 supplementation appear to be higher in our sample than that of a large cohort in Nordic countries [50]. Such healthy lifestyle behaviours have previously been suggested to modify the risk of disease progression in MS [3, 5–8, 53–55], indicating that future analyses of this dataset should enable important new findings on the effect of such risk-modifying behaviours on MS and its associated symptoms. 

The MS sample described appear to be a highly engaged and proactive group of patients. This is evidenced by the method of recruitment which was self-selecting from patients utilising online resources. The preliminary data suggests that a significant number in this sample have adopted lifestyle changes, much of which they would have learned about through self-directed learning beyond the clinical setting, and which demands a great deal of self-efficacy and commitment. A surprisingly large proportion of people continued to the end of the survey despite its considerable length. This was likely aided by the fact that participants could exit and return to the survey at their leisure. The large sample size and high item completion rate might indicate that the online MS community is motivated to contribute to novel research. Much of the feedback the research team received through the survey, emails, phone calls, and online comments was very positive. Participants expressed a desire for more research exploring lifestyle factors. Personal empowerment is key to successful adoption of healthful practices; people with MS who show increased levels of activation or self-efficacy also demonstrate positive changes in their self-management behaviours [56], self-reported improvements in health status [57], greater patient satisfaction, and improved quality of life [58].

Recruiting participants through web 2.0 platforms results in rapid access to a heterogeneous sample in a highly cost-effective way and may invite contribution from previously hidden populations [41]. Engagement with online communities is becoming increasingly common with the advent of online platforms such as PatientsLikeMe, which encourages people to share their health information for the benefit of others [59]. However, recruiting participants through online methods is not without challenges. Firstly, it is possible for people to conceal their identity. This can be helpful when conducting surveys because anonymity may encourage more honest responses and greater disclosure [60]. The disadvantage however is difficulty in verifying the diagnosis of participants in the absence of clinician or medical record confirmation. To improve accuracy of diagnosis in our study, questions were asked regarding diagnostic procedures, and an additional section was included for people with an unconfirmed diagnosis, such as clinically isolated syndrome (CIS). This enabled everyone to take part, whilst maintaining separate analysis. The NARCOMS registry also utilises self-enrolment, and a validation study confirmed a self-reported diagnosis of MS in 98.7% (SD ± 1.3) of cases [61]. This suggests that it is unlikely that people without a formal diagnosis of MS would take part in the study, although a validation study looking at a sample of the data would significantly improve the reliability of diagnosis. Survey completion rate was high; however, around 15% of participants dropped out at the contact details section. Removing the need for contact details might have improved the overall response rate; however, this would have prevented followup. It is also possible that there will be challenges associated with retention of study participants in follow-up surveys. Due to the international nature of the study and large sample size, only email reminders will be a feasible method of contact. Varied online engagement strategies will be necessary to maximise the response rate at followup. It is worth noting that many of the challenges associated with online surveys are similar to those that arise when conducting mail-outs of self-reporting questionnaires with large registries [60]. 

Although participants in 57 countries took part in the survey, the majority were residing in western countries where English is the primary language. This is likely a direct reflection of the sources of recruitment used by the researchers, which were mostly limited to English language, while the survey itself demanded a high level of literacy. Severe physical disability may have prevented some people with MS from taking part as they may not have been able to complete the survey without assistance. The fact that some participants were directly recruited through a website and associated forums promoting lifestyle modification may have resulted in the participation of individuals with an interest in, and more inclined to undertake, holistic disease management. As such, the findings from this study may not be generalisable to the global online MS community. 

When making a decision about which items to include in the survey, serious consideration was given to the time burden placed on participants, given that the survey intended to cover many different domains. Although validated tools were used where possible, and researcher-devised items were carefully constructed, with the survey piloted with a small group, there are a number of limitations to the survey. Firstly, the validated tools have not all been tested for validity and reliability across diverse MS samples. Different cultural understandings of health mean that the use of western terminology and concepts of health with a culturally diverse sample may have allowed bias. It is also unclear whether these validated tools have previously been tested in an online survey format. It is also possible that the study was more likely to select participants with a higher level of socioeconomic status due to the method of recruiting through web 2.0 platforms. Socioeconomic status of participants was not measured in the questionnaire. This is because of the international nature of the survey and difficulty correlating data across regions. 

A significant proportion of participants reported experiencing symptoms related to a recent relapse. It may be difficult for patients to distinguish between enduring relapse symptoms and a permanent progression of disability. This has the potential to impact other outcome variables, and future analyses will need account for this. All data were self-reported, which can lead to potential sources of bias due to over- or underreporting. This needs to be considered particularly in light of historic reporting of events which can be limited by poor recall, such as the number of relapses in the preceding five years, or poor knowledge, such as the number of lesions or vitamin D level. We were not able to verify the accuracy of these responses. Validation through medical records or physician's report would significantly increase the reliability of self-reported data. Consideration will be given to the limitations of data reliant on self-report that may be used in future analyses. Despite these limitations, some self-reported data in MS study participants have previously been found to be a reliable measure of outcomes [62, 63]. The importance of the patient perspective in measuring health outcomes has become, and continues to remain, a major focus of clinical and epidemiological research.

## 5. Conclusion

Increasingly, web 2.0 platforms are being embraced by patients and researchers alike, with the shared goal of improving patient-centred health outcomes. The characteristics described above suggest this online MS community recruited to our study is a heterogeneous and unique group who appear actively engaged and keen to contribute to research on health and lifestyle behaviour. Although there are several limitations to conducting research with online communities, this method provides efficient and rapid access to a large sample of people with MS. This study provides a sound platform to undertake exploratory analyses of lifestyle variables that have not previously been examined in such detail. The dataset will provide an important opportunity for novel research into lifestyle and health behaviours and their potential impact on MS outcomes over time. 

## Figures and Tables

**Table 1:** Summary of validated tools used.

Outcome variable	Instrument (reference)	Number of items	Authors (reference)
Disability	Patient determined disease steps (PDDS)	1	Hohol et al., 1995 [12]
Comorbidities	Self-administered comorbidity questionnaire (SCQ)	13	Sangha et al., 2003 [13]
Health-related quality of life	Multiple sclerosis quality of life-54 (MSQOL-54)	54	Vickrey et al., 1995 [14]
Dietary habits	Diet habits questionnaire (DHQ), modified	20	McKellar et al., 2008 [15]
Physical activity	International physical activity questionnaire (IPAQ)	7	Craig et al., 2003 [16]
Social support	Single item measure of social support (SIMSS)	1	Blake and McKay, 1986 [17]
Fatigue	Fatigue severity scale (FSS)	9	Krupp et al., 1989 [18]
Depression	Patient health questionnaire short version (PHQ-2)	2	Kroenke et al., 2003 [19]

**Table 2:** Classification of MS medication types.

First generation disease modifying drugs	Interferons
	Glatiramer acetate

Second generation disease modifying drugs	Alemtuzumab
	Cladribine
	Daclizumab
	Dimethyl fumarate
	Fingolimod
	Laquinimod
	Rituximab
	Teriflunomide
	Natalizumab

Chemotherapy or immunosuppressants	Azathioprine
	Cyclophosphamide
	Methotrexate
	Mitoxantrone
	Mycophenolate mofetil

IVIG or plasmapheresis	Immunoglobulins IVIG
	Plasmapheresis

Generic drugs	Low-dose naltrexone
	Minocycline

Steroids	Adrenocorticotropic hormone
	Prednisolone

Symptom modifying drugs	Baclofen
	Fampridine

**Table 3:** Characteristics of the study sample.

Characteristic	Number (%) (unless otherwise stated)
Gender	
Male	413/2318 (17.8)
Female	1905/2318 (82.2)
Age in 2012 (years)	
18–29	124/2443 (5.1)
30–39	621/2443 (25.4)
40–49	794/2443 (32.5)
50–59	656/2443 (26.9)
60–69	231/2443 (9.5)
>70	17/2443 (0.7)
Country of location	
USA	827/2518 (32.9)
Australia	649/2518 (25.8)
UK	416/2518 (16.5)
NZ	216/2518 (8.6)
Canada	107/2518 (4.2)
Other*	303/2518 (12.2)
Country of birth	
USA	790/2510 (31.5)
Australia	518/2510 (20.6)
UK	502/2510 (20.0)
NZ	174/2510 (6.9)
Canada	111/2510 (4.4)
Other^†^	415/2510 (16.5)
Marital status	
Married	1511/2475 (61.1)
Single	359/2475 (14.5)
Cohabitating/partnered	313/2475 (12.6)
Separated/divorced	262/2475 (10.6)
Widowed	30/2475 (1.2)
Family status	
No children	767/2477 (31.0)
One or more biological children	1644/2466 (66.7)
One or more step children	239/2466 (9.7)
No. of children^*∧*^, median (IQR)	2 (0–2)
Employment status	
Employed full time	807/2508 (32.2)
Employed part time	526/2508 (21.0)
Stay at home parent/carer	191/2508 (7.6)
Student full time	57/2508 (2.3)
Unemployed^‡^	203/2508 (8.1)
Retired due to age	79/2508 (3.1)
Retired due to medical reasons or disability	588/2508 (23.4)
Other	57/2508 (2.3)
Education status	
No formal schooling	3/2504 (0.1)
Primary school	55/2504 (2.2)
Secondary school	570/2504 (22.8)
Vocational training	405/2504 (16.2)
Bachelor's degree	897/2504 (35.8)
Postgraduate degree	574/2504 (22.9)

*Includes 72 other countries.

^†^Includes 52 other countries.

^*∧*^Includes biological and step children.

^‡^Collapsed from unemployed: seeking work/not seeking work.

**Table 4:** Diagnostic characteristics and level of disability.

	Number (%)
Number of years since diagnosis	
<1–5	1107/2459 (45.0)
6–10	577/2459 (23.5)
11–15	393/2459 (16.0)
16–20	186/2459 (7.6)
21–25	98/2459 (4.0)
26–30	48/2459 (2.0)
>30	50/2459 (2.0)
Disability (PDDS)	
Normal	734/2314 (31.7)
Mild disability	352/2314 (15.2)
Moderate disability	170/2314 (7.3)
Gait disability	373/2314 (16.1)
Early cane	265/2314 (11.5)
Late cane	175/2314 (7.6)
Bilateral support	139/2314 (6.0)
Wheelchair/scooter	103/2314 (4.5)
Bedridden	3/2314 (0.1)
Currently experiencing symptoms due to a recent relapse*	
Yes	643/2429 (26.5)
No	1455/2429 (59.9)
Unsure	331/2429 (13.6)

*Definition of relapse provided to participants.

**Table 5:** Diagnosed subtype of MS.

	Benign	Relapsing-remitting	Primary progressive	Secondary progressive	Progressive relapsing	Unsure/other
Type of MS first diagnosed with number (%)	99/2455 (4.0)	1880/2455 (76.6)	157/2455 (6.4)	47/2455 (1.9)	14/2455 (0.6)	258/2455 (10.5)
Type of MS currently diagnosed with number (%)	101/2460 (4.1)	1500/2460 (61.0)	184/2460 (7.5)	287/2460 (11.7)	47/2460 (1.9)	341/2460 (13.9)

**Table 6:** Relapse rate for relapsing-remitting participants.

	Self-diagnosed relapse rate			Doctor-diagnosed relapse rate		
	*n*	Mean (95% CI)	Median (IQR)	*n*	Mean (95% CI)	Median (IQR)
Number of relapses over last 12 months*	1475/1500	1.09 (1.01–1.17)	1 (0.0–2.0)	1456/1500	0.73 (0.67–0.78)	0 (0.0–0.1)
Number of relapses over last 5 years, annualised*	1409/1500	0.97 (0.92–1.03)	0.67 (0.4–1.2)	1399/1500	0.66 (0.62–0.70)	0.5 (0.2–1.0)

*Participants with relapsing-remitting MS.

**Table 7:** MSQOL summary scores.

Summary score	*n* (missing)	Distribution (Kolmogorov)	Mean (95% CI)	Median (IQR)
Overall quality of life subscore	2275 (244)	0.00	66.9 (66.1–67.7)	68.4 (55.0–81.7)
Physical health composite	1944 (575)	0.00	59.1 (58.1–60.0)	59.2 (42.3–77.2)
Mental health composite	2222 (297)	0.00	66.7 (65.8–67.6)	72.0 (51.2–84.1)

**Table 8:** Depression and fatigue screening.

	Cutoff	Positive, number (%, 95% CI)	Negative, number (%, 95% CI)
Depression screen (PHQ-2)	Negative < 3; positive ≥ 3	431/2231 (19.3, 17.7–21.0)	1800/2231 (80.7, 79.0–82.3)
Fatigue (FSS) mean score	Negative < 4; positive ≥ 4	1408/2143 (65.7, 63.7–67.7)	735/2143 (34.3, 32.3–36.3)

**Table 9:** Comparison of data with NARCOMS and MSBase registries.

	HOLISM	NARCOMS [22]	MSBase [45]
Female (%)	82.2	74.9	71.5
Age at diagnosis (years)	37.0 (median)	36.9 (mean)	32.2 (mean)
Age at symptom onset (years)	31.0 (median)	32.4 (mean)	Data not provided
Age at enrolment (years)	46.0 (median)	42.8 (mean)	42.7 (mean)
Disease duration at enrolment (years)	6.0 (median)	Data not provided	10.4 (mean)

**Table 10:** Country of residence.

Country	*n*
Armenia	1
Australia	649
Austria	2
Belgium	5
Brazil	7
Bulgaria	1
Canada	107
China	1
Croatia	7
Cyprus	1
Czech Republic	2
Denmark	10
Estonia	2
Finland	4
France	11
Gibraltar	2
Guam	1
Germany	29
Greece	12
Iceland	2
India	3
Indonesia	1
Iran	1
Ireland	36
Israel	2
Italy	6
Kuwait	1
Lebanon	1
Luxembourg	1
Malta	2
Mexico	3
Namibia	1
Netherlands	28
New Zealand	216
Norway	13
Philippines	2
Poland	2
Portugal	6
Puerto Rico	3
Qatar	2
Romania	4
Russian Federation	2
Saudi Arabia	2
Serbia	2
Singapore	2
Slovakia	4
Slovenia	2
South Africa	30
Spain	9
Sweden	17
Switzerland	8
Syria	1
Trinidad and Tobago	1
Turkey	1
United Arab Emirates	4
United Kingdom	416
United States	827
